# Supplementary material for: Allosteric regulation and crystallographic fragment screening of SARS-CoV-2 NSP15 endoribonuclease
Source: Nucleic Acids Res. 2023 Apr 28;51(10):5255–70. doi: 10.1093/nar/gkad314 (PMC10250223; doi:10.1093/nar/gkad314)
Supplement: gkad314_Supplemental_Files [file gkad314_supplemental_files.zip › Supplementary datasets.zip!/183/Refine_4/01-BUSTER/Cycle-1/shell.01/R_Rfree.html]

# Rxpct factors in resolution bins at cycle 0


|  |  |  |  |  |  |  |  |  |
| --- | --- | --- | --- | --- | --- | --- | --- | --- |
|  | **<d> (Å)**  Rxpct\_Work | Rxpct\_Free | Rxpct\_All | |  |  |  |  |
|  | 14.0455 | 0.2992 | 0.3179 | 0.2999 |  |
|  | 8.1092 | 0.2180 | 0.2359 | 0.2190 |  |
|  | 6.2814 | 0.2619 | 0.2428 | 0.2610 |  |
|  | 5.3087 | 0.2078 | 0.1953 | 0.2072 |  |
|  | 4.6818 | 0.1687 | 0.1727 | 0.1689 |  |
|  | 4.2349 | 0.1690 | 0.1503 | 0.1680 |  |
|  | 3.8955 | 0.1811 | 0.1834 | 0.1812 |  |
|  | 3.6265 | 0.1897 | 0.1994 | 0.1902 |  |
|  | 3.4065 | 0.2045 | 0.1940 | 0.2040 |  |
|  | 3.2223 | 0.1906 | 0.2037 | 0.1913 |  |
|  | 3.0650 | 0.1949 | 0.1861 | 0.1945 |  |
|  | 2.9287 | 0.1961 | 0.1927 | 0.1959 |  |
|  | 2.8091 | 0.2041 | 0.2110 | 0.2044 |  |
|  | 2.7031 | 0.2092 | 0.2146 | 0.2095 |  |
|  | 2.6082 | 0.2269 | 0.2248 | 0.2268 |  |
|  | 2.5227 | 0.2323 | 0.2410 | 0.2328 |  |
|  | 2.4450 | 0.2435 | 0.2525 | 0.2439 |  |
|  | 2.3741 | 0.2678 | 0.2482 | 0.2669 |  |
|  | 2.3091 | 0.2841 | 0.3078 | 0.2851 |  |
|  | 2.2491 | 0.3110 | 0.3025 | 0.3107 |  |
|  | **Overall** | 0.2089 | 0.2071 | 0.2088 |  |

# Rxpct factors in resolution bins at cycle 1


|  |  |  |  |  |  |  |  |  |
| --- | --- | --- | --- | --- | --- | --- | --- | --- |
|  | **<d> (Å)**  Rxpct\_Work | Rxpct\_Free | Rxpct\_All | |  |  |  |  |
|  | 14.0455 | 0.2979 | 0.3156 | 0.2986 |  |
|  | 8.1092 | 0.2167 | 0.2349 | 0.2177 |  |
|  | 6.2814 | 0.2602 | 0.2409 | 0.2593 |  |
|  | 5.3087 | 0.2063 | 0.1960 | 0.2059 |  |
|  | 4.6818 | 0.1688 | 0.1719 | 0.1690 |  |
|  | 4.2349 | 0.1683 | 0.1537 | 0.1675 |  |
|  | 3.8955 | 0.1810 | 0.1860 | 0.1813 |  |
|  | 3.6265 | 0.1900 | 0.2004 | 0.1905 |  |
|  | 3.4065 | 0.2030 | 0.1916 | 0.2024 |  |
|  | 3.2223 | 0.1894 | 0.2024 | 0.1902 |  |
|  | 3.0650 | 0.1927 | 0.1840 | 0.1923 |  |
|  | 2.9287 | 0.1949 | 0.1892 | 0.1947 |  |
|  | 2.8091 | 0.2037 | 0.2172 | 0.2043 |  |
|  | 2.7031 | 0.2083 | 0.2171 | 0.2088 |  |
|  | 2.6082 | 0.2262 | 0.2253 | 0.2261 |  |
|  | 2.5227 | 0.2317 | 0.2386 | 0.2321 |  |
|  | 2.4450 | 0.2435 | 0.2491 | 0.2438 |  |
|  | 2.3741 | 0.2689 | 0.2522 | 0.2681 |  |
|  | 2.3091 | 0.2865 | 0.3208 | 0.2881 |  |
|  | 2.2491 | 0.3103 | 0.2997 | 0.3099 |  |
|  | **Overall** | 0.2082 | 0.2073 | 0.2081 |  |

# Rxpct factors in resolution bins at cycle 2


|  |  |  |  |  |  |  |  |  |
| --- | --- | --- | --- | --- | --- | --- | --- | --- |
|  | **<d> (Å)**  Rxpct\_Work | Rxpct\_Free | Rxpct\_All | |  |  |  |  |
|  | 14.0455 | 0.2986 | 0.3132 | 0.2992 |  |
|  | 8.1092 | 0.2171 | 0.2299 | 0.2178 |  |
|  | 6.2814 | 0.2560 | 0.2501 | 0.2557 |  |
|  | 5.3087 | 0.2070 | 0.2050 | 0.2069 |  |
|  | 4.6818 | 0.1694 | 0.1746 | 0.1697 |  |
|  | 4.2349 | 0.1704 | 0.1498 | 0.1693 |  |
|  | 3.8955 | 0.1810 | 0.1758 | 0.1808 |  |
|  | 3.6265 | 0.1889 | 0.2060 | 0.1897 |  |
|  | 3.4065 | 0.2012 | 0.1944 | 0.2008 |  |
|  | 3.2223 | 0.1900 | 0.2033 | 0.1907 |  |
|  | 3.0650 | 0.1941 | 0.1899 | 0.1939 |  |
|  | 2.9287 | 0.1933 | 0.1855 | 0.1930 |  |
|  | 2.8091 | 0.1995 | 0.2181 | 0.2003 |  |
|  | 2.7031 | 0.2046 | 0.2005 | 0.2043 |  |
|  | 2.6082 | 0.2236 | 0.2260 | 0.2237 |  |
|  | 2.5227 | 0.2244 | 0.2310 | 0.2248 |  |
|  | 2.4450 | 0.2350 | 0.2436 | 0.2354 |  |
|  | 2.3741 | 0.2565 | 0.2501 | 0.2562 |  |
|  | 2.3091 | 0.2723 | 0.3092 | 0.2739 |  |
|  | 2.2491 | 0.3171 | 0.3070 | 0.3167 |  |
|  | **Overall** | 0.2068 | 0.2067 | 0.2068 |  |

# Rxpct factors in resolution bins at cycle 3


|  |  |  |  |  |  |  |  |  |
| --- | --- | --- | --- | --- | --- | --- | --- | --- |
|  | **<d> (Å)**  Rxpct\_Work | Rxpct\_Free | Rxpct\_All | |  |  |  |  |
|  | 14.0455 | 0.2955 | 0.2948 | 0.2955 |  |
|  | 8.1092 | 0.2101 | 0.2275 | 0.2112 |  |
|  | 6.2814 | 0.2407 | 0.2517 | 0.2412 |  |
|  | 5.3087 | 0.1980 | 0.2006 | 0.1982 |  |
|  | 4.6818 | 0.1651 | 0.1787 | 0.1659 |  |
|  | 4.2349 | 0.1666 | 0.1605 | 0.1662 |  |
|  | 3.8955 | 0.1768 | 0.1917 | 0.1775 |  |
|  | 3.6265 | 0.1862 | 0.2182 | 0.1878 |  |
|  | 3.4065 | 0.1923 | 0.2012 | 0.1928 |  |
|  | 3.2223 | 0.1844 | 0.1986 | 0.1852 |  |
|  | 3.0650 | 0.1890 | 0.1935 | 0.1892 |  |
|  | 2.9287 | 0.1873 | 0.2015 | 0.1879 |  |
|  | 2.8091 | 0.1913 | 0.2177 | 0.1925 |  |
|  | 2.7031 | 0.1976 | 0.1983 | 0.1976 |  |
|  | 2.6082 | 0.2150 | 0.2275 | 0.2157 |  |
|  | 2.5227 | 0.2169 | 0.2282 | 0.2176 |  |
|  | 2.4450 | 0.2270 | 0.2417 | 0.2277 |  |
|  | 2.3741 | 0.2444 | 0.2456 | 0.2444 |  |
|  | 2.3091 | 0.2684 | 0.2894 | 0.2694 |  |
|  | 2.2491 | 0.3095 | 0.3056 | 0.3093 |  |
|  | **Overall** | 0.2004 | 0.2097 | 0.2008 |  |
